# Does joint mobilization effectively improve chronic ankle instability ? A systematic review and meta-analysis

**DOI:** 10.3389/fmed.2025.1711920

**Published:** 2026-01-14

**Authors:** Qimei Jiang, Xiaoping Zhou, Rubing Yan, Chuansheng Hong, Peng Tang, Shaopeng Wu, Hongbing Li, Xiaoyu Wu, Qinghua Luo

**Affiliations:** Department of Rehabilitation, Southwest Hospital, Third Military Medical University (Army Medical University), Chongqing, China

**Keywords:** joint mobilization, chronic ankle instability, systematic review, meta-analysis, randomized controlled trials

## Abstract

**Objective:**

The aim of this study is to systematically evaluate the effectiveness of joint mobilization (JM) in improving chronic ankle instability (CAI).

**Methods:**

We conducted a search in six databases—PubMed, Embase, Cochrane Library, Web of Science, Pedro, and China National Knowledge Infrastructure (CNKI)—up to March 10, 2025. We included all published randomized controlled trials (RCTs) on JM for the treatment of CAI. The primary outcome measure was ankle function (Cumberland Ankle Instability Tool, CAIT), and the secondary outcomes were pain and ankle range of motion (ROM). All outcome measures were analyzed by calculating standardized mean differences (SMDs) and 95% confidence intervals (CIs).

**Results:**

We identified 237 studies across six databases and ultimately included 8 RCTs involving 318 subjects with CAI. Our findings indicate that, compared to the control group, JM effectively improves ankle function (SMD = 1.62; 95% CI = 0.29–2.94; *P* = 0.02, *I*^2^ = 92%) and ROM (SMD = 1.18; 95% CI = 0.22–2.15; *P* < 0.0001, *I*^2^ = 90%) in CAI subjects, but no significant difference was observed in ankle pain (SMD = –0.27; 95% CI = –0.68 to 0.13; *P* = 0.18, *I*^2^ = 0%).

**Conclusion:**

JM has been shown to effectively improve ankle joint function and ROM in subjects with CAI, although it does not significantly alleviate pain associated with CAI.

## 1 Introduction

Chronic ankle instability (CAI) is a common musculoskeletal disorder that occurs after repeated ankle sprains, and after developing CAI, the ankle joint becomes less stable and more prone to sprains (1). CAI affects a large percentage of the population, and previous studies have shown that more than 20% of patients with acute lateral ankle sprains eventually develop CAI (2). CAI often results in pain, swelling, decreased or absent proprioception due to ankle sprains, as well as decreased muscle strength and atrophy of the lower extremities if prolonged immobilization occurs, which can be a serious complication in daily life, work, and school (3, 4). More seriously, CAI subjects may lead to joint degeneration as well as reduced quality of life, consuming greater medical resources (5).

The etiology of CAI is varied and multifactorial, and is mainly categorized into mechanical instability of bones and joints such as ligamentous laxity or joint overuse and sensory deficits such as impaired proprioception and impaired neuromuscular control (6). The inability to accurately assess the etiology of the disorder leads to more complex and time-consuming rehabilitation of CAI, requiring multiple interventions over time.

At the present stage, the clinical treatment methods of CAI mainly include conservative treatment and surgical treatment. Conservative treatment mainly refers to physical therapy, such as muscle strength training and proprioceptive training (7). In addition, the use of some adjuncts, such as ankle braces and elastic bands can provide some support during exercise, and the use of non-steroidal anti-inflammatory drugs (NSAIDs) can help control pain and inflammation (8). Surgeries like ligament reconstruction can restore stability to the ankle joint when functional deficits or pain are severe (9). However, these treatments have limitations. Physical therapy methods require long-term adherence and are not effective in subjects with severe or chronic instability (10). Supports and elastic bands provide stabilization temporarily are and do not address the underlying mechanical or neuromuscular deficits fundamentally, much less fully restore proprioceptive problems (11). Surgical treatment, although effective in severe subjects, is accompanied by risks of postoperative infection, anesthetic complications, and a longer recovery period (12).

In recent years, new rehabilitation techniques, particularly JM, have been continuously updated and have garnered increasing attention in various musculoskeletal disorders. JM is a passive manual manipulation technique where rehabilitation therapists, after assessment, apply passive joint movements in specific directions with appropriate force and frequency to restore normal joint range of motion, thereby helping to restore stability while quickly alleviating pain (13, 14). For CAI subjects, JM can help improve the bony structural position and joint space of the ankle joint, as well as alleviate neuromuscular issues, thereby enhancing joint stability, proprioception, and overall ankle joint function (15). These therapeutic effects of JM can further reduce the incidence of ankle sprains and enhance subjects’ daily activity capabilities and athletic performance.

Despite the growing interest in JM as a therapeutic option for CAI and the various clinical trials and observational studies conducted by peers, the results of the studies at this stage are varied. A number of studies have shown that JM reduces pain and improves joint function well, whereas a bit of research has found little or no effect (16, 17). In addition, the specific JM therapy and assessment tools used in the studies vary widely, which further makes the evaluation of efficacy controversial.

Based on the results of different studies at this stage, and in order to better explore the comprehensive effects of JM on CAI subjects, we conducted the present systematic evaluation and meta-analysis with the aim of comprehensively evaluating existing studies, and by analyzing the results of the objective data from existing randomized controlled trials (RCTs), we hope to obtain more intuitive and evidence-based conclusions from the studies, and to provide theoretical basis for the treatment of CAI by JM in the clinical setting.

## 2 Materials and methods

### 2.1 Protocol and registration

This study was conducted strictly following the PRISMA guidelines (18). Before the official commencement of this research, we completed the registration on the PROSPERO platform, registration number: CRD420251015918. The data for this systematic review and meta-analysis were sourced from formally published relevant RCT studies and did not involve the direct recruitment of subjects or the collection of any original patient data. Therefore, ethical and moral approval was not required.

### 2.2 Search strategy

The search process for this study was conducted independently by two researchers. The databases searched included PubMed, Embase, Cochrane Library, Web of Science, Pedro, and China National Knowledge Infrastructure (CNKI), with the search period extending from the inception of the databases until March 10, 2025. No language or regional restrictions were applied during the search. The search terms included “joint mobilization,” “chronic ankle instability,” and related free terms. A manual search of relevant databases was also performed to ensure that all pertinent literature was included. After both researchers completed their independent searches, a third researcher cross-checked the search results and strategies to ensure consistency. The relevant search records and search strategies are provided in Supplementary Material 1.

### 2.3 Study selection

All identified literature was imported into EndNote (Version X9, Clarivate Analytics) for initial screening and filtering. The screening process was also conducted independently by two researchers, who objectively assessed the studies based on predefined inclusion and exclusion criteria (19). Initially, duplicate studies were removed. Then, the titles and abstracts were reviewed to exclude studies that were irrelevant or had incompatible research designs. Finally, after reviewing the full text, the final inclusion of studies was determined. After both researchers completed their independent screening, a third researcher conducted a review. In cases of discrepancies, a meeting was held to reach a consensus on the final inclusion of studies.

The inclusion criteria for this study were as follows: (1) Only subjects with CAI were included, diagnosed based on the clinical diagnostic standards defined by the “Diagnostic Criteria for CAI.” (2) Intervention: Various types of JM. (3) Comparison group: No intervention, placebo, or corresponding intervention group with JM + conventional treatment (ensuring JM is the single variable). (4) At least one of the following outcomes: primary outcome: Ankle function [CAIT(Cumberland Ankle Instability Tool)], secondary outcomes: pain [VAS(Visual analog scale)], and ankle range of motion (ROM). (5) Study design: RCTs.

Exclusion criteria: (1) Studies with incomplete data. (2) Data presented only in graphical form. (3) Intervention group includes joint mobilization as part of combined therapy, but JM is not the single variable compared to the control group. (4) Randomized crossover study design.

### 2.4 Data extraction and management

The information extracted from the studies ultimately included in the analysis includes: study authors, publication year, country where the study was conducted, age of participants in both the intervention and control groups, the interventions used in both groups, intervention duration and frequency, and outcome measures. The extraction of this basic information was carried out independently by two researchers, following a predefined template for data entry. Once completed, the data was reviewed by a third researcher for comparison and verification. In cases of discrepancies, all three researchers reviewed the original texts together to reach a consensus.

### 2.5 Quality assessment

The risk of bias in this study was assessed using Review Manager 5.4. The assessment was conducted independently by two authors, who each read the full text of the included studies and evaluated them objectively. After the individual assessments were completed, a third researcher cross-checked the two results, and the final outcome was determined after discussion among the three researchers. The assessment considered the following seven aspects: Random sequence generation (selection bias), Allocation concealment (selection bias), Blinding of participants and personnel (performance bias), Blinding of outcome assessment (detection bias), Incomplete outcome data (attrition bias), Selective reporting (reporting bias), and Other bias. Each aspect was rated as one of the following three categories: (1) Low risk of bias, (2) high risk of bias, or (3) unclear risk of bias.

### 2.6 Data analysis

To evaluate the outcomes in each study, the difference between baseline and post-intervention values was calculated to determine the effect of the intervention compared to the control group. This meta-analysis was performed using Review Manager 5.4 software, developed by the Cochrane Collaboration. For continuous outcomes, such as Cumberland Ankle Instability Tool (CAIT) scores, the standardized mean difference (SMD) along with its 95% confidence interval (CI) was calculated. For dichotomous outcomes, such as adverse events, risk ratios (RRs) with corresponding 95% CIs were computed. Heterogeneity between the groups was assessed using Cochran’s Q statistic and the *I*^2^ test (20). A fixed-effect model was used when no significant heterogeneity was detected (Q test *P* > 0.05 or *I*^2^ < 50%). In contrast, when significant heterogeneity was present (*P* < 0.05 or *I*^2^ > 50%), a random-effects model was applied. If *I*^2^ exceeded 50%, sensitivity analysis or subgroup analysis was conducted to explore the potential sources of heterogeneity. Statistical significance was defined as a *P* < 0.05.

## 3 Results

### 3.1 Search results

**FIGURE 1:**
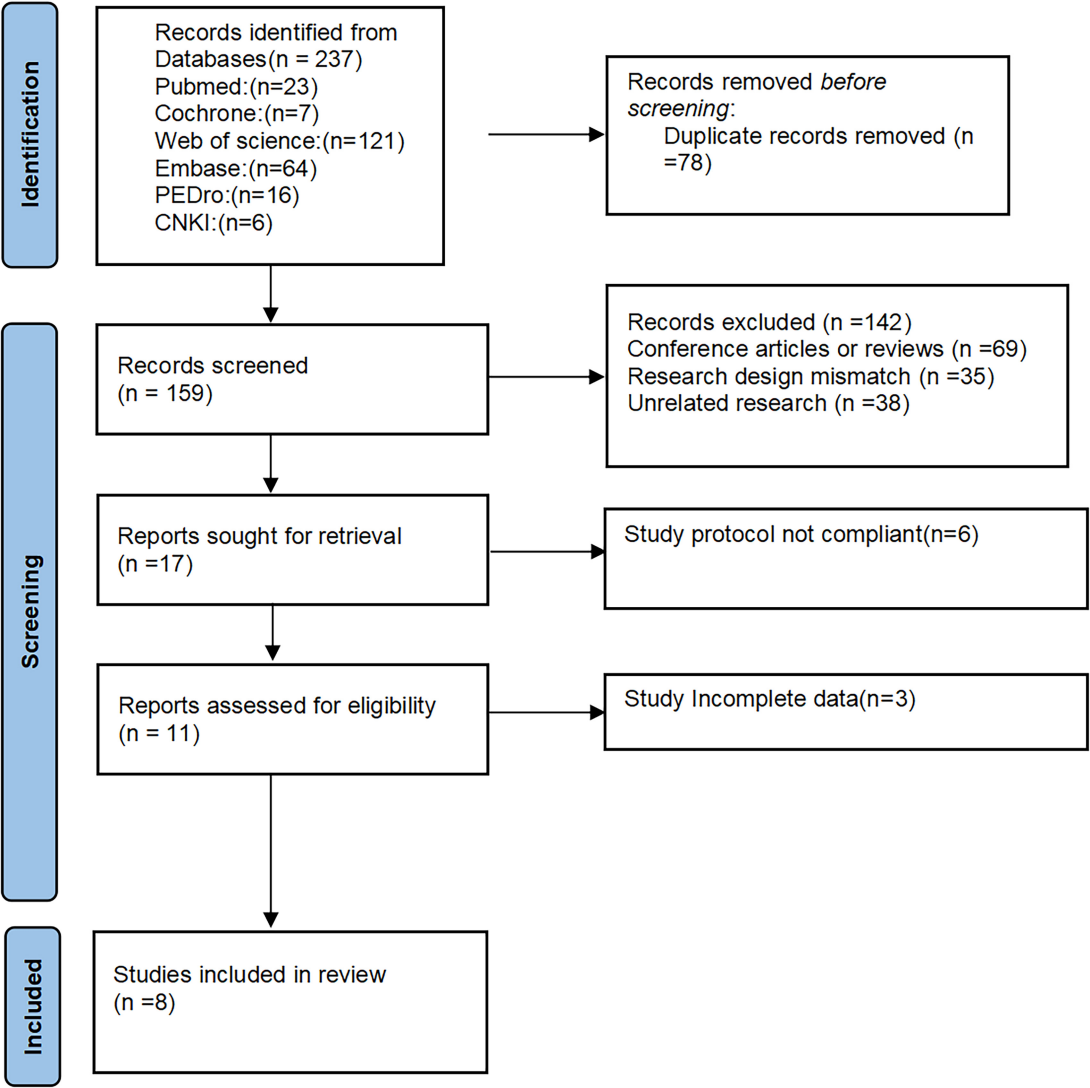
Flow diagram of included studies.

The literature search and selection process is shown in Figure 1. In this study, we conducted a search across six commonly used medical databases and identified 237 relevant studies (PubMed = 23, Cochrane = 7, Web of Science = 121, Embase = 64, PEDro = 16, CNKI = 6). All references were imported into Endnote for management. Initially, 78 duplicate entries were excluded, leaving 159 studies. Subsequently, 69 conference papers or reviews, 35 studies with mismatched study designs, and 38 irrelevant studies were excluded, resulting in 17 studies for full-text review. After reading the full texts, 6 studies with research protocols that did not meet the requirements and 3 studies with incomplete data were excluded. Finally, 8 studies were included for analysis.

### 3.2 Study characteristics

Table 1 provides a detailed description of the basic characteristics of the included studies. A total of 8 RCTs published between 2012 and 2024 were included, involving 318 subjects with CIA. Seven studies were published in English (21–27), and one study was published in Chinese (28). Four studies were conducted in China (24, 26–28), two in the United States (21, 23), and the remaining two in Spain (22, 25). Among the 8 studies, 4 compared JM with no treatment (21–24), 3 compared JM plus conventional treatment with conventional treatment alone (26–28), and the last study compared JM with a placebo treatment (25). Four of the studies assessed the CAIT (22, 24, 27, 28), 6 evaluated ankle ROM (21–25, 27), and only 2 studies assessed pain (26, 27). Although different assessment scales were used, data related to the same outcome measures were analyzed together.

**TABLE 1:** Basic characteristics of included citations.

References	Country	E/C(N)	Age (year) (M ± SD)	Experimental group	Control group	JM type	Time/times/weeks of yoga	Outcome
Tang et al. (26)	China	32/31	E:28.69 ± 3.57C:28.69 ± 3.57	JM+conventional rehabilitation	conventional rehabilitation	Maitland	40 min/3/w/8 w	VAS
	Spain	18/15	E:65.5 ± 5.6 C:63.7 ± 8.9	JM	Placebo	–	Once	ROM
Yin et al. (27)	China	16/16	E:20.00 ± 1.58C:20.31 ± 0.75	JM + routine rehabilitation	Routine rehabilitation	Maitland	10 min/3/w/4 w	CAIT,VAS,ROM
Beazell et al. (21)	USA	15/13	E:25.2 ± 8.2 C:23.8 ± 5.6	JM	No treatment	–	2 min/1/w/3 w	ROM
	Spain	30/29	E:26.48 ± 4.03 C:26.48 ± 4.03	JM	No treatment	MWM	Two sets/2/w/3 w	CAIT,ROM,
Harkey et al. (23)	USA	15/15	E:21.5 ± 3.4 C:20.8 ± 2.0	JM	No treatment	Maitland	Once	ROM
Lao et al. (24)	China	17/20	E:19.8 ± 1.37 C:22 ± 1.78	JM	No treatment	Maitland	10 min/3/w/4 w	CAIT,ROM
Dong et al. (28)	China	18/18	E:27.94 ± 3.08 C:28.05 ± 2.90	JM+Functional motor therapy	Functional motor therapy	–	60 min/7/w/4 w	CAIT

E, experimental group; C, control group; JM, joint mobilization; CAIT, Cumberland Ankle Instability Tool; VAS, visual analog scale; ROM, range of motion.

### 3.3 Quality assessment

**FIGURE 2:**
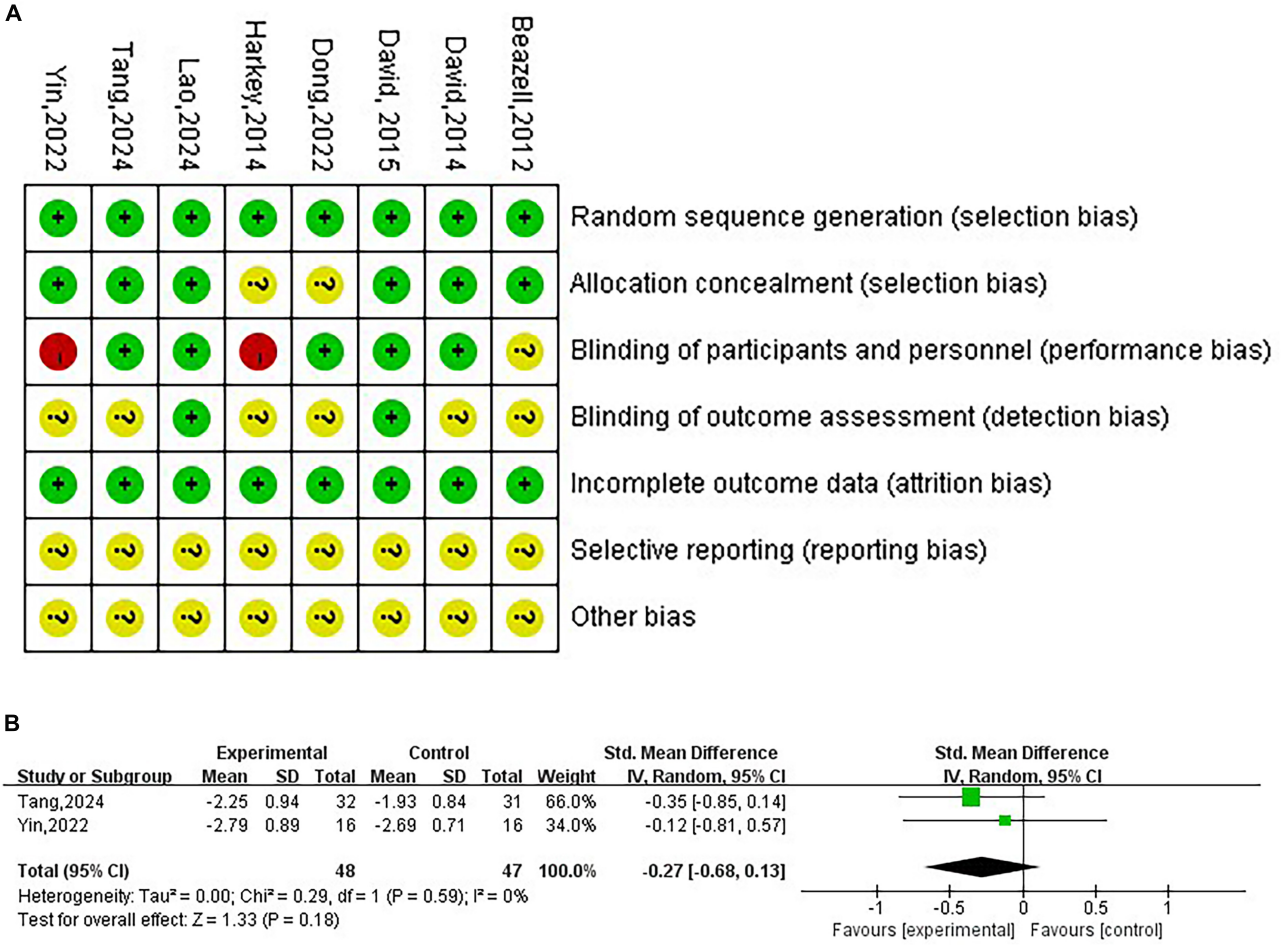
**(A)** Risk of bias graph. **(B)** Risk of bias summary.

The risk of bias results for the included studies are shown in Figures 2A, B. Only RCTs were included, and therefore, all eight studies (100%) employed randomization. Six studies (75%) provided a detailed description of the allocation concealment method (21, 22, 24–27), while the other two did not mention whether allocation concealment was used (23, 28). Five studies (62.5%) employed blinding for both participants and investigators (22, 24–26, 28), with two studies using single blinding (23, 27), and one study did not specify whether blinding was used (21). Two studies (25%) implemented blinding for outcome assessors and data analysts (24, 25), while the remaining six studies did not indicate whether blinding was applied to these individuals (21–23, 26–28). All included studies reported complete data (100%), which was one of our inclusion criteria. None of the studies clearly stated the risk of selection bias (100%). Given the small number of studies included in this analysis, we did not conduct a publication bias analysis.

#### 3.3.1 Analysis of ankle joint function

**FIGURE 3:**
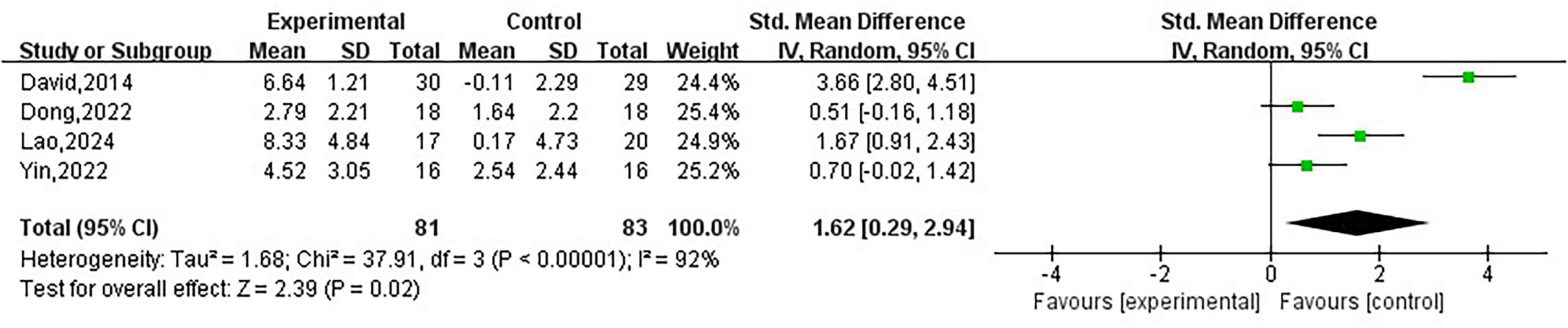
Analysis of ankle joint function.

A total of four studies (22, 24, 27, 28) used the CAIT to assess changes in ankle function in subjects with CAI before and after treatment, including 164 subjects, and the results are shown in Figure 3. The analysis revealed that, compared to the control group, JM effectively improved ankle function in subjects with CAI (SMD = 1.62; 95% CI = 0.29–2.94; *P* = 0.02, *I*^2^ = 92%). Due to high heterogeneity, we conducted a sensitivity analysis and found that after excluding two studies (22, 24), JM still significantly improved ankle function in CAI subjects, with a substantial reduction in heterogeneity (SMD = 0.6; 95% CI = 0.11–1.08; *P* = 0.02, *I*^2^ = 0%).

#### 3.3.2 Analysis of ankle ROM

**FIGURE 4:**
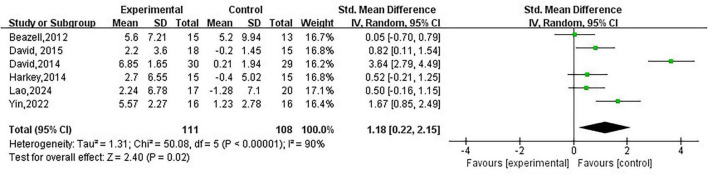
Analysis of ankle ROM.

A total of six studies (21–25, 27) evaluated changes in ankle range of motion in subjects with CAI before and after treatment, including 219 subjects, as shown in Figure 4. The analysis revealed that, compared to the control group, JM significantly improved ankle range of motion in CAI subjects (SMD = 1.18; 95% CI = 0.22–2.15; *P* < 0.0001, *I*^2^ = 90%). Due to high heterogeneity, we conducted a sensitivity analysis and found that after excluding two studies (22, 27), JM still effectively improved ankle range of motion in CAI subjects, with a substantial reduction in heterogeneity (SMD = 0.48; 95% CI = 0.12–0.83; *P* = 0.008, *I*^2^ = 0%).

#### 3.3.3 Analysis of ankle pain

**FIGURE 5:**
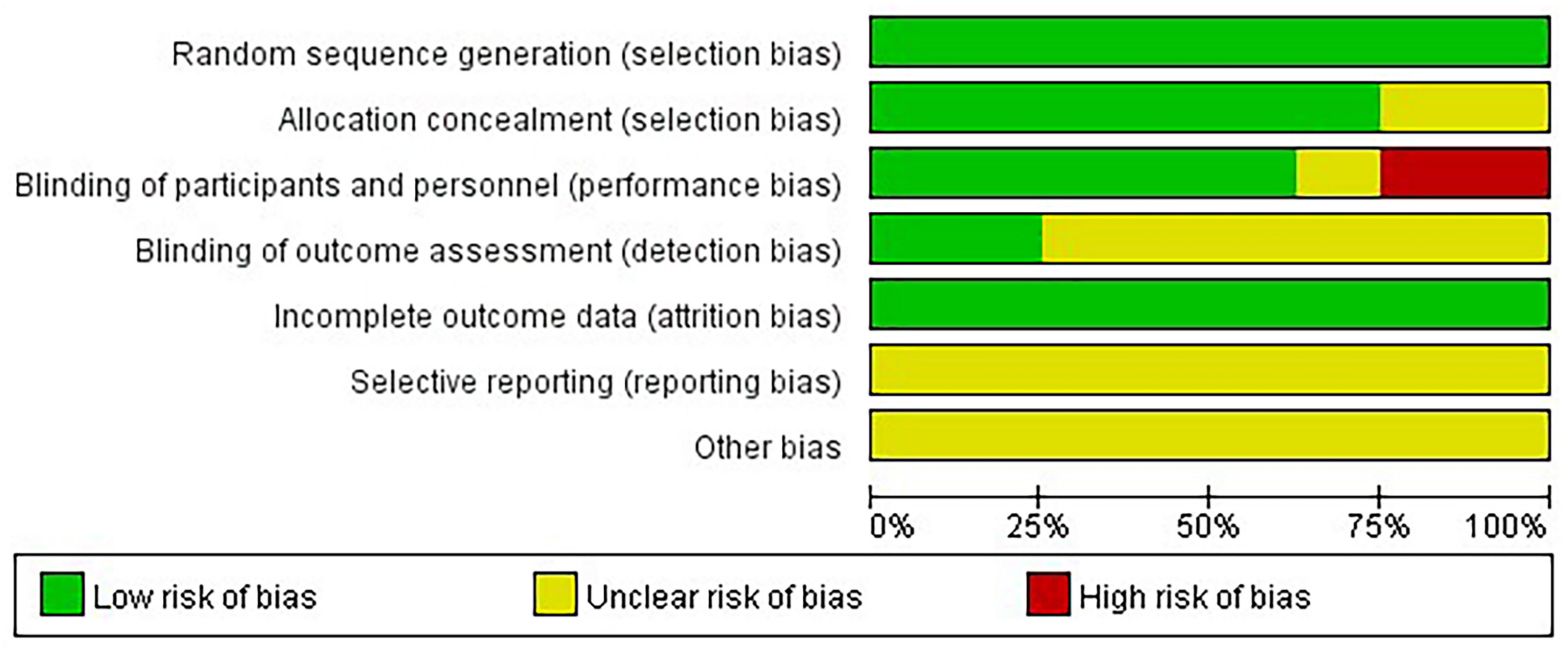
Analysis of ankle pain.

Two studies (26, 27) used the VAS to evaluate changes in ankle pain in CAI subjects before and after treatment, as shown in Figure 5. The analysis indicated that JM did not result in a significant difference in the improvement of ankle pain compared to the control group (SMD = –0.27; 95% CI = –0.68 to 0.13; *P* = 0.18, *I*^2^ = 0%).

## 4 Discussion

This meta-analysis included a total of 8 relevant RCT studies to evaluate the effects of JM on ankle function, ROM, and pain in CAI subjects. We did not alter the research protocol after registration.

Our results indicate that, compared to the control group (which received placebo therapy, no treatment, or conventional treatment), JM effectively improved ankle joint function and ROM in subjects with CAI. However, there was no significant difference in pain relief between the JM and control groups. These findings are generally consistent with previous studies, although some differences were observed. The improvement in ankle joint function and ROM with JM aligns with earlier research (29). Interestingly, a 2019 study on the duration of JM therapy for functional recovery in CAI subjects found that more than six interventions were required for significant improvement in ankle joint function (30). Additionally, another meta-analysis on shoulder instability showed that JM had no significant effect on improving shoulder pain (31).

### 4.1 The effect of JM on ankle joint function

The results of this study indicate that JM can effectively improve ankle joint function in CAI subjects compared to the control group. However, due to the high heterogeneity of the results, we conducted a sensitivity analysis to explore the sources of heterogeneity. The sensitivity analysis found that after excluding one study with the shortest intervention duration and another study with the highest dropout rate (22, 24), the results remained unchanged, but the heterogeneity of the results was significantly reduced.

JM can effectively improve ankle joint function in CAI subjects, potentially through the following mechanisms. These mechanisms primarily include enhancing proprioception in CAI subjects, increasing ankle joint stability, and promoting muscle function recovery (32, 33). These three primary mechanisms facilitate the rapid restoration of ankle joint physiological function and can assist CAI subjects in rebuilding their motor control system, thereby promoting functional recovery.

First, JM plays a crucial role in the recovery of proprioception, particularly for CAI. Frequent ankle sprains can lead to proprioceptive impairment, which in turn affects joint stability and motor control capabilities (34). JM achieves this by directly adjusting the ankle joint and its surrounding soft tissue structures, stimulating receptors and sensors within the joint, particularly the smaller, often overlooked joint receptors. These receptors transmit more proprioceptive information to the cerebral cortex, maintaining stability during both rest and movement (35, 36). This enhanced signaling or feedback helps strengthen neuromuscular control and maintain joint stability.

In terms of enhancing ankle joint stability, JM can effectively improve subjects’ ability to perceive changes in joint position, thereby strengthening CAI subjects’ control over movement. This enhanced stability is essential, as it can effectively prevent recurrent ankle sprains caused by proprioceptive deficits (37). This benefit of JM may be related to neuromuscular reflex pathways, where mechanical stimuli and pressure applied to the ankle joint are transmitted to ankle receptors, peripheral muscle spindles, and tendons (38). These stimuli activate afferent nerves, which transmit signals to the spinal cord. The spinal cord then relays these signals to the cerebral cortex and motor areas, thereby participating in joint localization and movement control.

At a deeper level, JM can modulate multiple signaling pathways associated with proprioception and motor control, such as the Wnt/β-catenin pathway, Notch signaling pathway, and PI3K/Akt pathway, among others (39). The expression of these signaling pathways can improve ankle joint stability and effectively reduce anxiety and feelings of insecurity during movement. For CAI subjects, the fear of sprains is a common psychological activity, and each ankle injury may lead to further ligament laxity or worsening functional impairment, ultimately resulting in joint instability (40). This instability not only affects ankle joint function but also impacts subjects’ psychology, leading to a lack of confidence in movement or reduced performance (41). Following JM therapy, ligament and joint stability in CAI subjects is partially restored. Research indicates that JM not only enhances joint stability but also significantly reduces anxiety caused by instability, improving subjects’ anticipation and satisfaction with physical activity, thereby enhancing overall performance (42).

JM also plays a crucial role in restoring neuromuscular function in CAI subjects. During ankle instability, muscle control around the ankle decreases, leading to delayed reactions and muscle weakness. After JM therapy, joint gliding ability improves, surrounding soft tissue tension decreases, and certain neural pathways are activated, thereby accelerating muscle reaction speed and enhancing overall coordination (43). When manually stretching the muscles and ligaments around the joint, JM can also promote local blood circulation, increase muscle blood supply, thereby improving muscle endurance and function around the ankle joint, and aiding in the restoration of joint dynamic stability. This process helps improve ankle joint movement control and enhances the performance of CAI subjects during high-intensity activities or rapid movements (44).

### 4.2 The effect of JM on ankle ROM

The results of this study indicate that JM can effectively improve ankle joint ROM in CAI subjects. However, the analysis results showed significant heterogeneity, so we further conducted a sensitivity analysis to explore the sources of heterogeneity. After a detailed comparison of the study designs and other aspects of the six analyzed studies, we found that only one study (27) combined JM therapy with traditional rehabilitation training, while the other studies used JM intervention alone. It can be speculated that the synergistic effect of combined therapy may lead to greater improvements in ankle joint ROM, resulting in some differences from studies that used JM alone, thereby contributing to the high heterogeneity of the results. Additionally, another study (25) used placebo treatment as the control group, while other studies compared with no treatment at all; the placebo may have had a psychological effect. After excluding these two studies, we found that heterogeneity significantly decreased without altering the results, indicating that differences in study design and control groups have a substantial impact on heterogeneity.

Ankle instability and frequent sprains are common clinical symptoms in CAI subjects, particularly during physical activities. During movement, CAI subjects experience significant joint stiffness, especially during ankle dorsiflexion and internal/external rotation (45). Restricted ROM can also affect daily activities and quality of life, and may increase the risk of future ankle sprains worsening. However, compared to protective immobilization, long-term restriction of ankle joint movement or even inactivity can lead to increased tension or stiffness in the surrounding soft tissues (such as ligaments and tendons), further reducing ROM. Upon removal of immobilization, subjects may face issues such as muscle atrophy and impaired balance function (46). Prolonged immobilization and reduced ROM may also disrupt the circulation of synovial fluid within the joint, exacerbating joint stiffness and instability.

JM employs relatively gentle manual techniques, including rolling, sliding, separation, and traction, to reduce tension in the soft tissues surrounding the joint, enhance joint gliding ability and rotational range, and effectively expand ROM (47). The mechanism may involve JM effectively promoting synovial fluid flow, enhancing cartilage lubrication, and reducing intra-articular adhesions or stiffness (48). Joint mobilization techniques also have a regulatory effect on inflammatory factors within the joint. Through mechanical stimulation, JM can promote the release of bioactive molecules such as serotonin and prostaglandins within the joint, which play a crucial role in alleviating inflammatory responses. Reduced inflammation further alleviates edema and aids in the recovery of joint ROM (49). JM can also stimulate fibroblasts and chondrocytes to synthesize more collagen, which aids in the repair of damaged areas within the joint, gradually restoring the ankle joint to its normal morphology and thereby increasing ankle joint ROM (50).

From a biomechanical perspective, the role of JM is not limited to expanding the range of motion of the joint; it also comprehensively regulates the joint and its surrounding tissues. JM can promote the joint to complete normal physiological movements, restore many physiological functions of the joint to normal levels, enhance the stability and flexibility of the joint, and thereby improve the rehabilitation outcomes for CAI subjects (51).

### 4.3 The effect of JM on ankle pain

The research findings show that JM has no significant effect on alleviating the pain of CAI subjects. CAI can cause local chronic pain and may lead to inflammatory reactions in the joints. However, the research data indicate that the intensity of chronic pain in CAI subjects is at a relatively low level (52). This situation may be related to the subjects’ own pain tolerance, pain perception mechanisms, and adaptive changes induced by chronic pain. The development of chronic pain often accompanies plastic changes in the nervous system, and subjects gradually get accustomed to this pain, which weakens the perception of pain, and this again affects the evaluation of efficacy (53). The pathological anatomical condition of CAI is relatively complex, involving factors such as joint mechanical instability, ligament injury, and insufficient muscle control. These factors may have complex associations with the degree and nature of pain (54). Even though JM may have some beneficial effects on physiological levels, its direct impact on pain relief is not as prominent as imagined.

As mentioned earlier, the therapeutic mechanisms of JM may include reducing intra-articular pressure, releasing soft tissues around the ankle joint, and improving local blood circulation. JM employs moderate, gentle joint manipulation techniques to alleviate tissue compression caused by ankle joint instability, thereby alleviating inflammatory reactions around the ankle joint (55, 56). However, despite the objective theoretical basis for these mechanisms, the data from this study indicate that JM has no significant effect on pain relief, which is worth discussing. This may be closely related to factors such as limited existing research, short intervention duration, short treatment duration, insufficient frequency, or individual differences.

To know, few studies consider pain as the primary outcome measure, which may be one of the reasons for our negative conclusions. Most studies on CAI focus on aspects such as functional recovery and optimization of joint range of motion, and pain is often considered a secondary outcome. Therefore, pain assessment may not fully reflect the potential of arthritis to alleviate pain in CAI subjects. Clinically, there are significant differences in pain perception among individuals, and factors such as mental state, pain sensitivity, and treatment expectations will also affect the results of pain evaluation (57).

Although our study results indicate that JM has no significant effect on pain in CAI subjects, this does not rule out potential benefits. To further explore the role of JM in improving pain in CAI subjects, future clinical studies may require more scientifically rigorous study designs, including large-scale, multi-center randomized controlled trials to ensure accuracy, and the integration of objective data such as biomarkers in blood or synovial fluid to assess the efficacy of JM. Additionally, future studies should consider the impact of individual differences on treatment outcomes, including whether to stratify subjects into subgroups based on age, gender, medical history, and different severity levels of CAI, or to conduct studies targeting specific populations.

## 5 Limitations

This study objectively evaluated the impact of JM on CAI subjects, but it also has some limitations. First, there are currently limited RCT studies on JM therapy for CAI. Although this study endeavored to ensure that JM was the sole variable, some of the included studies involved combination therapy, and the synergistic effects could not be assessed or quantified. As more RCTs are published in the future, this issue may be resolved. Second, the treatment duration and follow-up period in the included RCTs were approximately 4 weeks, but there was inconsistency across all studies. We had planned to conduct subgroup analyses based on different intervention durations during this study, but due to insufficient data, this analysis could not be performed.

## 6 Conclusion

JM has been shown to effectively improve ankle joint function and ROM in subjects with CAI, although it does not result in a significant reduction in pain. Despite this, JM is recommended as an effective clinical treatment for CAI, especially in improving joint function and preventing further episodes of instability.

## Data Availability

The original contributions presented in the study are included in the article/Supplementary material, further inquiries can be directed to the corresponding author.
